# Hazard Screening Methods for Nanomaterials: A Comparative Study

**DOI:** 10.3390/ijms19030649

**Published:** 2018-02-25

**Authors:** Barry Sheehan, Finbarr Murphy, Martin Mullins, Irini Furxhi, Anna L. Costa, Felice C. Simeone, Paride Mantecca

**Affiliations:** 1 Department of Accounting and Finance, University of Limerick, V94PH93 Limerick, Ireland; finbarr.murphy@ul.ie (F.M.); martin.mullins@ul.ie (M.M.); irini.furxhi@ul.ie (I.F.); 2 Institute of Science and Technology for Ceramics (CNR-ISTEC), National Research Council of Italy, Via Granarolo 64, 48018 Faenza (RA), Italy; anna.costa@istec.cnr.it (A.L.C.); felice.simeone@istec.cnr.it (F.C.S.); 3 Department of Earth and Environmental Sciences, Particulate Matter and Health Risk (POLARIS) Research Centre, University of Milano Bicocca, 20126 Milano, Italy; paride.mantecca@unimib.it

**Keywords:** nanomaterials, hazard assessment, Bayesian network, weight of evidence, multi-criteria decision analysis, human health hazard screening

## Abstract

Hazard identification is the key step in risk assessment and management of manufactured nanomaterials (NM). However, the rapid commercialisation of nano-enabled products continues to out-pace the development of a prudent risk management mechanism that is widely accepted by the scientific community and enforced by regulators. However, a growing body of academic literature is developing promising quantitative methods. Two approaches have gained significant currency. Bayesian networks (BN) are a probabilistic, machine learning approach while the weight of evidence (WoE) statistical framework is based on expert elicitation. This comparative study investigates the efficacy of quantitative WoE and Bayesian methodologies in ranking the potential hazard of metal and metal-oxide NMs—TiO_2_, Ag, and ZnO. This research finds that hazard ranking is consistent for both risk assessment approaches. The BN and WoE models both utilize physico-chemical, toxicological, and study type data to infer the hazard potential. The BN exhibits more stability when the models are perturbed with new data. The BN has the significant advantage of self-learning with new data; however, this assumes all input data is equally valid. This research finds that a combination of WoE that would rank input data along with the BN is the optimal hazard assessment framework.

## 1. Introduction

Hazard identification is a primary step in the risk assessment of engineered nanomaterials (NM) [1,2]. Four decades have passed since Norio Taniguchi first coined the term “nanotechnology” [3], and hazard assessment remains a continuous research effort to support the development and commercialization of nanomaterials [4]. A consensus acceptance of hazard and risk assessment methodologies is essential in order to agree on accepted risk reduction measures for NM [5]. Effective risk communication between stakeholders is necessary for the sustainable growth of the nanotechnology industry [6]. Notwithstanding this, the rapid commercialisation of nano-enabled products continues to out-pace the development of a prudent risk management mechanism that is accepted by the scientific community and enforced by regulators. The good news is that a growing body of academic literature is contributing to the development of increasingly accurate quantitative risk assessment methods, but a validated, replicable and transparent hazard identification tool remains elusive. This paper represents a valuable addition to this literature set as it seeks to identify suitable methodologies to contend with the complex nature of NM hazard identification.

Recently, Bayesian methodologies have been gaining support in the context of NM risk assessment [7,8,9,10], whilst more established weight of evidence (WoE) based frameworks have been criticized for being overly reliant on expert judgement and qualitative data [11]. There remain, however, significant deficiencies and inconsistencies in key experimental results required to facilitate conclusive risk management decision-making [4,12,13]. An intermediate approach of appending scientific expert opinion to real-world NM physico-chemical, biological, and toxicological data to determine NM hazard potential has offered some degree of success. Both Bayesian networks (BN) and quantitative WoE methods have been proposed as effective frameworks in achieving this task [9,14]. This paper offers a timely comparison of both methodologies allowing for a meaningful comparison of both results and performance.

Hazard screening (or ranking) is a method used to benchmark the intrinsic hazard potential of several NMs against one another [15]. Expensive and time-consuming toxicological testing has resulted in a concentration of focus towards specific NMs. Relative hazard screening can therefore be used to read-across experimentally demonstrated adverse effects for a specific NM to one with similar physico-chemical characteristics and little experimental evidence in terms of hazard potential. Through this benchmarking approach, proactive risk management may be inferred by enforcing occupational exposure limits (OEL) for NMs akin to their relative hazard score.

BNs are probabilistic hierarchical models that, given a dataset, express probabilistic causal relationships (i.e., conditional probabilities) between the different parameters [16]. The chain of influences between parameters can be rendered graphically by linking nodes (i.e., parameters) by one-way directed links that determine the nature of the causal dependencies. Each individual node has a finite set of mutually exclusive states, with each state described by a probabilistic expression determined by empirical relationships, mechanistic descriptions, or expert judgement [17]. BN probabilistic models are suited to NM hazard identification through their ability to capture heterogeneous datasets that may contain missing, or conflicting, information. The model is particularly suited to problems with limited data through its ability to iteratively refine forecasts as new information becomes available. The NM hazard ranking tool proposed by Marvin et al. [9] applied Bayesian network (BN) construction, parameterisation, and uncertainty analysis to metal and metal-oxide NMs. This BN tool showed high accuracy, with 72% hazard prediction precision in an out-of-sample test.

WoE represents a diverse collection of methods used to synthesise and evaluate individual lines of evidence (LOE) to form a conclusion [14,18]. WoE approaches have been classified by the degree of quantitative criteria incorporated to deduct decisions [19]. These methods range from basic qualitative assessment in the form of listing evidence to fully quantitative procedures which include statistical methods or multi-criteria decision analysis (MCDA) [19]. Hristozov et al. [14] developed the first quantitative MCDA approach for human health hazard screening of NMs, and illustrated the approach using a nano-TiO_2_ case study. A logic WoE methodology was complemented with quantitative MCDA to produce a “hazard score” for nano-TiO_2_ which may be compared to those of other nanomaterials for hazard ranking.

In this article, a quantitative WoE with MCDA framework was applied for metal and metal-oxide NMs—TiO_2_, Ag, ZnO. The resulting hazard rankings were compared to those demonstrated via the BN application in Marvin et al. [9]. The results of both methods were also tested for sensitivity to input variables, and the validation of results was demonstrated for the BN and examined for the WoE method. The sources of information are the same for both the quantitative WoE framework and the BN allowing for a comparative analysis. This is the first study that compares the relative hazard rankings of NMs using separate assessment tools with the same reference literature. This is also the first application of the quantitative WoE tool used to rank the hazard potential of several NMs.

## 2. Materials and Methods

This article examines the BN hazard ranking tool made available and described by Marvin et al. [9]. Furthermore, the quantitative WoE with MCDA methodology is replicated from Hristozov et al. [14]. Hence, a concise account of both model formulations is presented in this section. Detailed descriptions are provided where the methodologies are adapted or extended for the purposes of this comparative analysis.

A quantitative WoE with multi-criteria decision analysis (MCDA) hazard ranking model is demonstrated for TiO_2_, Ag and ZnO. The resultant hazard ranking of the WoE is contrasted to that of the BN constructed in Marvin et al. [9] in both normal and stressed states by means of sensitivity and uncertainty analysis. The sensitivity of the hazard potential for each NM to input variables is investigated for both BN and WoE methods. The accuracy of the hazard prediction is tested with a cross-validation analysis.

### 2.1. Data

Marvin et al. [9] gathered physico-chemical and toxicity data of metal and metal-oxide NMs from studies reported in the scientific literature in the period of 2009–2015. In total, 32 scientific articles were used resulting in 559 cases or “lines of evidence” (LOE) containing data which may influence the hazard potential of NMs. For the purposes of this comparative analysis, the literature for TiO_2_, Ag, and ZnO are investigated due to their prolificacy in the database. This represents 48% (or 225 cases) of the total data.

For the quantitative WoE method, 26 of the 32 peer-reviewed articles were analysed with respect to the information provided on physico-chemical properties, toxicity, and data quality as per the REACH requirements [20]. The 6 remaining papers were omitted from the analysis because they did not reference the NMs being examined. The next sections detail the methods used to construct and evaluate both the BN and quantitative WoE hazard ranking tools. The full list of literature is provided in Appendix A.

### 2.2. Bayesian Network Methodology

The process of building a BN consists of three steps: (i) node (or variable) identification, (ii) establish directed links for a causal network, and (iii) determine the conditional probability tables (CPTs) [16]. In the context of NM hazard assessment, the most relevant physico-chemical characteristics and biological effects are selected as nodes via expert elicitation processes. Furthermore, the initial causal structure and parameterisation of the CPTs is determined by two rounds of expert consultation. Using the 559 cases derived from the literature data, the expectation-maximization machine learning algorithm is used to further refine and optimize the causal structure and conditional probabilities of the BN. The Hugin 8.5 software is used to construct and learn the BN.

For the purposes of this paper, the validation of the BN and sensitivity analysis is performed specifically with respect to TiO_2_, Ag, and ZnO. To test the hazard prediction accuracy of the BN tool, an out-of-sample test is carried out against 41 cases omitted from the network structure and parameterisation learning procedure. This comprised of inputting the physico-chemical parameters of each case as evidence into the BN and comparing the predicted NM hazard (the most likely state with the highest % probability) to the true value of observed NM hazard determined from the literature.

Two methods of sensitivity analysis are performed on the BN. The first of which is a value of information analysis, which uses the entropy function to measure the sensitivity of the hypothesis variable, NM hazard, to the other nodes within the BN [21,22]. The entropy H(X) (measure of randomness) of a discrete random variable X is defined as:

(1) $$H\left(X\right)=-\sum_{X}P\left(X\right)\cdot \log P\left(X\right),$$

where P(X) is the probability distribution of *X*.

This analysis ranks the physico-chemical properties, administration route, and study type variables in order of influence on the NM hazard node. Next, a scenario analysis is performed to assess the sensitivity of the order of hazard ranking to changes in NM physico-chemical characteristics.

### 2.3. Quantitative Weight of Evidence Methodology

Following the methodology of Hristozov et al. [14], the hazard of each LOE is evaluated based on three criterion: NM physico-chemical properties, toxicity, and data quality. Each study is considered a single LOE unless multiple experimental results are observed. The model follows a Logic method, where each LOE is evaluated according to a set decision steps comprehensively described in Hristozov, Zabeo, Foran, Isigonis, Critto, Marcomini and Linkov [14], and briefly summarised below.

1. LOE index values based on physico-chemical properties: Physico-chemical criterion (BET surface area, primary particle size, aspect ratio, surface coating, ζ-potential, purity, composition, bioaccumulation) are evaluated according a state-specific scoring system in the [0,100] range. These discretised states, or classes, refer to the segregation of the criteria into their components of increased/decreased hazard (i.e., aspect ratio ≥ 1:3 = high hazard = 100; aspect ratio < 1:3 = low hazard = 25). The LOE-specific index $S_{j}^{p.chem}$ is subsequently determined by the arithmetic mean of each score given to $c_{1,\left(j\right)}^{p.chem}$, …, $c_{n,\left(j\right)}^{p.chem}$.
2. LOE index values based on toxicity: Five hazard classes ($C_{i}^{tox}$) of increasing evidence of toxicity to humans according to US EPA guidelines are specified and mapped onto a scoring system within the [0,100] range [23]. Specific rules apply for the study, or LOE, to be categorised into a specific class. For example, for class $C_{5}^{tox}=100$, there must be convincing causal evidence between the NM and biological effect. LOE may fall into one or more classes based on the conclusions provided by the author. Hence, a percentage $D_{i,j}$ would be assigned according to the likelihood the conclusions fit into a certain class. The LOE-specific index value $S_{j}^{tox}$ is then calculated by the following equation:
  (2) $$S_{j}^{tox}=\sum_{i=1}^{5}C_{i}^{tox}\;D_{i,j}$$
3. Total LOE index values: The LOE indices for physico-chemical data and toxicity are aggregated to form a global LOE index ($S_{j}$) representing intrinsic hazard demonstrated by the study. Since both do not have equal weight in the hazard assessment, a weighted sum (WS) operator is applied. The weights $w_{p.chem}$ < $w_{tox}$ imply that toxicity evidence explains more about the intrinsic hazard potential of a NM than physico-chemical evidence. The following equation illustrates the aggregation of the indices:
  (3) $$S_{j}=S_{j}^{p.chem}w_{p.chem}+S_{j}^{tox}w_{tox}.$$
4. LOE weight: The weight ($W_{j}$) of each LOE is established according to a Logic model that uses regulatory data quality criteria (adequacy, reliability, statistical power, toxicological significance) to infer the study’s relevance to measuring the hazard potential of a NM [20]. Each weight is normalised by dividing them by their total sum:
  (4) $$w_{j}^{\prime }=\frac{W_{j}}{\sum_{\forall j}W_{j}}.$$
5. Weighted LOE index value: The impact of each LOE on the total hazard assessment is calculated by obtaining the product of the global LOE index value ($S_{j}$) and normalised study quality weight ($w_{j}^{\prime }$):
  $$WI_{j}=S_{j}w_{j}^{\prime }$$

The sum of each weighted LOE index value represents the hazard score (V) for the NM, which can be compared to hazard scores computed for other NM for relative hazard ranking.

(5) $$V=\sum_{j=1}^{n}\;WI_{j.}$$

Hazard scores were calculated for TiO_2_, Ag, and ZnO using steps 1–5 and ranked accordingly. Monte Carlo analysis was used to probabilistically assess the sensitivity of the hazard scores to the weights applied to the physico-chemical, toxicity, and study quality criteria. This consists of random sampling from the distribution of $S_{j}^{p.chem}$, $S_{j}^{tox}$, and/or $W_{j}$ in a finite number of simulations to derive a distribution of results (V′). The variability of the distribution of results provides information on the uncertainty inherent to the WoE methodology, and the sensitivity of the of the input parameters to the hazard ranking of the three NMs.

The sensitivity and uncertainty analysis comprised of the following steps:

- The probability distributions for the input criterion were set at the full range of the normalisation scale, that is, [0,100] for $S_{j}^{p.chem}$ and $S_{j}^{tox}$ and [0,1] for $W_{j}$.
- Four sampling scenarios were investigated. Three of which involved sampling input values of one of the criterion ($S_{j}^{p.chem}$, $S_{j}^{tox}$, $W_{j}$) from their probability distributions while holding the others constant. The fourth sampled input values from the probability distributions of all three criterion. The sampling was uniformly distributed within the interval.
- Each sampling scenario was simulated 10,000 times and the total weighted LOE index value $V_{i}^{\prime }$ recorded at each iteration.

## 3. Results

### 3.1. Hazard Ranking of Nanoparticles Composed of TiO_2_, Ag and ZnO

**Figure 1:**
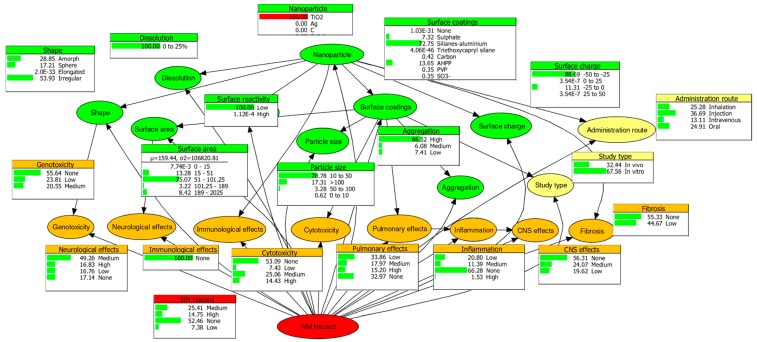
Graphical structure and parameterization of the Bayesian networks (BN) with TiO_2_ as the sample nanomaterial (NM). Ellipses represent nodes and directed links signify the conditional relationship between parent and child nodes. The accompanying bar charts denote the % state probabilities. The nodes are colour categorised into green for physicochemical properties, yellow for experimental methodology, orange for biological effects, and red for NM hazard potential. Adapted from Marvin et al. [9].

**Figure A1:**
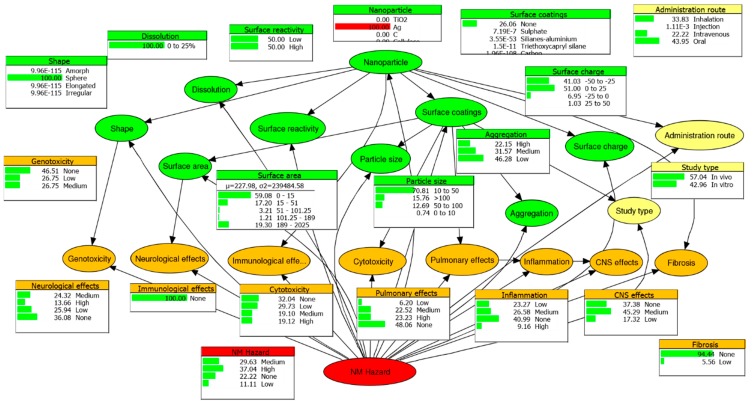
Graphical structure and parameterization of the BN with Ag input as evidence. Ellipses represent nodes and directed links signify the conditional relationship between parent and child nodes. The accompanying bar charts denote the % state probabilities. The nodes are colour categorised into green for physicochemical properties, yellow for study type, orange for biological effects, and red for NM hazard potential. Adapted from Marvin et al. [9].

**Figure A2:**
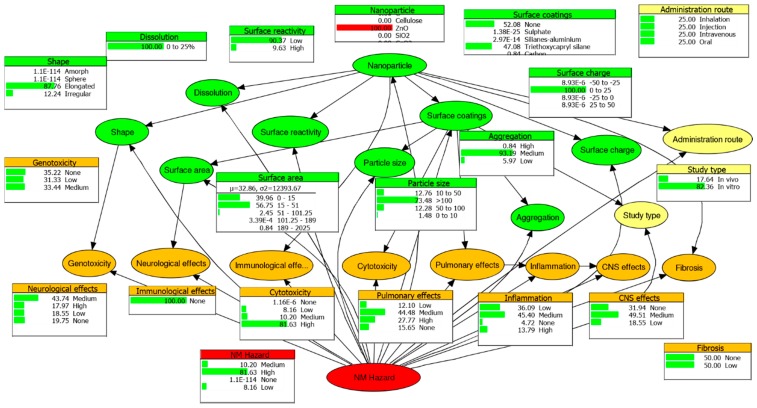
Graphical structure and parameterization of the BN with ZnO input as evidence. Ellipses represent nodes and directed links signify the conditional relationship between parent and child nodes. The accompanying bar charts denote the % state probabilities. The nodes are colour categorised into green for physicochemical properties, yellow for study type, orange for biological effects, and red for NM hazard potential. Adapted from Marvin et al. [9].

Figure 1 illustrates the graphical structure and parameterisation of the BN with TiO_2_ as the sample NM. This shows the marginal probability of each state within the nodes and their causal linkages resulting from an expert elicitation process as well as structure and parameter machine learning. The NM hazard node (Figure 1, red ellipse) represents the “hazard potential” of TiO_2_, implying the probability of no, low, medium, and high hazard is 52.46, 7.38, 25.41, and 14.75% respectively. To obtain a normalised variable for the purposes of hazard ranking, the weighted sum operation of the NM hazard state probabilities and a uniform scale [$0,\;\frac{1}{3},\;\frac{2}{3},\;1$] was applied to acquire a normalised hazard score of 34%. The uniform scale represents the increasing hazard potential of the states “None”, “Low”, “Medium”, and “High”. The same method was used to probabilistically characterise the hazard of Ag and ZnO (see Appendix, Figure A1 and Figure A2). The normalised hazard scores led to hazard potentials, ranked from highest to lowest, of ZnO (91%), Ag (61%), TiO_2_ (34%).

The quantitative WoE with MCDA methodology was applied to the same literature evidence used to train the BN in order to produce a total weighted index value (V), representing the intrinsic hazard potential of the NM. The application of the framework to the TiO_2_ literature is provided in Table 1 and explained below. The results for TiO_2_ is provided in Table 1, with representations for Ag and ZnO are supplied in the Appendix C and Appendix D, Table A1 and Table A2 respectively.

The first step of the method required the expert evaluation of the physico-chemical data according to the index scoring system described in the methodology section. The aggregated LOE-specific score based on physico-chemical properties ranged from 30.56 to 61.11 with an average value of 41.54.

Each LOE was subsequently evaluated according to toxicological evidence, resulting in scores ranging from 0 to 87.50 with an average of 43.18. These LOE indices were aggregated by a weighted sum operator to form a global LOE index ($S_{j}$), representing the intrinsic hazard potential inferred from each study. The contribution of each LOE to the concluding hazard score is regulated by means of the study quality weighting procedure. This facilitates the inclusion of a heterogenous evidence base, attributing higher weights to studies most relevant to hazard assessment. The product of the LOE-specific index value ($S_{j}$) and the normalised study quality weight ($w_{j}^{\prime }$) determines the weighted LOE-specific hazard score ($WI_{j}$) for study j. The scores are summed into the total weighted index value of 44.24 (V), the hazard score of TiO_2_. This WoE methodology was applied to the Ag and ZnO literature (see Appendix C and Appendix D), producing hazard scores of 45.26 and 52.34 respectively. Hence, the relative hazard ranking of each NM from highest to lowest according to their hazard score (V): ZnO (52.34), Ag (45.26), TiO_2_ (44.24). The WoE model workings are provided in Excel format in the Supplementary Material.

### 3.2. Evaluation of the Performances of Bayesian Networks (BN) and WoE

An out-of-sample, or cross-validation, test was used to evaluate the prediction accuracy of NM hazard by the BN. This procedure involved applying the input parameters (physico-chemical data, study type, administration route) for each case as evidence and observing the probability distribution amongst the states of the hypothesis node, NM hazard. The NM hazard state (None, Low, Medium, High) with the highest likelihood was chosen as the “predicted” state, which was compared to the state observed in the literature.

A total of 43 TiO_2_, Ag, and ZnO cases that were not used in the structure and parameter learning procedure of the BN were used in the cross-validation analysis by Marvin et al. [9] and examined individually for the purposes of this paper. Table 2 illustrates the results of the cross-validation test for 15 sample cases, showing that NM hazard is accurately predicted in 9 out of 15 cases. The prediction accuracy for all 40 cases is 67%.

Out of the 43 cases analysed in the cross-validation test, 24 were TiO_2_, 10 Ag, and 9 ZnO. The prediction accuracy by NM type shows 100% for ZnO, 70% for Ag and 54% for TiO_2_. The low precision for TiO_2_ was investigated further, and it was observed that the results may be skewed due to a repetition of a study which produced varying levels of observed NM hazard with the same input parameters. With these cases omitted, 67% of TiO_2_ cases are predicted correctly. The full cross-validation analysis is available in the Supplementary Material.

The evaluation of the performance of quantitative WOE, which is, strictly speaking, not a prediction model, but an approach used to inform decision-making based on the strength of evidence, relied on uncertainty and sensitivity analysis.

### 3.3. Sensitivity and Uncertainty Analysis of BN and WoE

An out-of-sample, or cross-validation, test was used to evaluate the prediction accuracy of NM hazard by the BN. This procedure involved applying the input parameters (physico-chemical data, study type, administration route) for each case as evidence and observing the probability distribution amongst the states of the hypothesis node, NM hazard. The NM hazard state (None, Low, Medium, High) with the highest likelihood was chosen as the “predicted” state, which was compared to the state observed in the literature. 

A value of information (VOI) analysis was applied to the BN to analyse the potential usefulness of additional information (input nodes) to the hypothesis variable, NM hazard. The task of the VOI analysis is to identify, using entropy reduction, the variables which are most informative with respect to the hypothesis variable [21]. Entropy reduction calculated the degree to which the input variables (physico-chemical properties, administration route, and study type) influenced the NM hazard node. A higher value indicates a higher sensitivity of NM hazard to the corresponding input node.

The results of the VOI analysis are presented in Table 3. Study type (0.34), particle size (0.28), and surface coatings (0.26) are distinguished as properties that have significant influence over the NM hazard node for TiO_2_. In contrast, administration route (0.64), surface coatings (0.53), and surface charge (0.37) have the largest effect for the Ag hazard node. The entropy of the input parameters on the NM hazard node for ZnO showed the least significance, with surface reactivity (0.16) being the only meaningful result. Marvin et al. (2017) demonstrated that cytotoxicity evidence also has a highly influential effect on the hazard potential of TiO_2_, Ag, and ZnO [9].

The sensitivity of individual input parameters on the NM hazard node may also be analysed for the BN. The effect of evidence from each state for the physico-chemical input parameters particle size (Table 4) and surface area (Table 5) on the predicted hazard potential is observed. The results include the normalised NM hazard potential as discussed before. Table 4 illustrates that the hazard ranking with no evidence (from highest to lowest: ZnO, Ag, TiO_2_) remains consistent with one exception, when particle size is within the range 0 nm to 10 nm. In this case TiO_2_ becomes the highest hazard (100%), followed by ZnO (86%), and Ag (50%). In contrast, Table 5 shows that the original hazard ranking order is true for only the surface area of between 189 and 2025 m^2^/g. The four other states of surface area still rank ZnO with the highest hazard potential, then TiO_2_ and finally Ag.

A Monte Carlo analysis was applied to the quantitative WoE with MCDA methodology to evaluate the model in terms of uncertainty of the final hazard ranking. In the literature, Monte Carlo analyses have been used to analyse the sensitivity of decision criteria to input variables for quantitative WOE models [14,37]. This approach allows for an examination of the influence of the input variables on the total weighted index value (*V*) for each NM. Four sampling scenarios were performed:

1. Vary LOE-specific index of physico-chemical properties ($S_{j}^{p.chem}$), while keeping all other input parameters constant.
2. Vary LOE-specific index of toxicity ($S_{j}^{tox}$), while keeping all other input parameters constant.
3. Vary the study quality weights ($W_{j}$), while keeping all other input parameters constant.
4. Vary all input parameters $S_{j}^{p.chem}$, $S_{j}^{tox}$, and $W_{j}$

Descriptive statistics of the resulting probability distributions of $V_{i}^{\prime }$ each of the four sampling scenarios are illustrated in Table 6. The metrics used to illustrate the influence of the variation of the input parameters on the observed hazard score (*V*) are the mean, standard deviation, and average absolute deviation of $V_{i}^{\prime }$ (for i=1:10,000). The absolute deviation is calculated as [14]:

(6) $$\Delta V_{i}=\left|V_{i}^{\prime }-V\right|\;in\;\left[0,100\right]$$

The average $\Delta V_{i}$ is low for sampling scenarios (i) and (iii), increasing slightly for scenario (ii), and increasing substantially for scenario (iv) where the average absolute deviation is 6.5% for TiO_2_, 6.3% for Ag, and 10.3% for ZnO when all the input parameters are considered uncertain. The analysis indicates that hazard score produced by the WoE model is least sensitive to changes in the study quality weight parameters, and influenced most by changes to the index of toxicity.

The uncertainties attributed to the WoE methodology originate from the expert elicitation methods utilized to determine the indexes, metrics, and criterion in the initial model formation, and also in the interpretations of the expert appraising each study. The stability of the final hazard ranking order was assessed to ensure that the order is a function of intrinsic hazard associated with the NMs, or simply the output of model noise. To evaluate the stability of the hazard ranking order, within each sampling scenario the results $V_{i}^{\prime }$ (i=1:10,000) for each NM were ranked. There are six possible permutations for the hazard ranking order of the three NMs (see Table 7). For example, under sampling scenario (ii) the index of toxicity ($S_{j}^{tox}$) is varied resulting in simulated $V_{i}^{\prime }$ for TiO_2_, Ag, and ZnO. This results in 30,000 simulations in total (i.e., 10,000 $V_{i}^{\prime }$ for each NM). Each simulation i was ranked according to the hazard scores $V_{i}^{\prime }$ calculated for each NM.

Table 7 illustrates that the observed hazard ranking order (from lowest to highest: (a) TiO_2_, Ag, ZnO) is stable across the four stressed scenarios in 44% of all samples. The order remains consistent to the observed order (a) in 55% of simulations where the physico-chemical index was varied, in 22% of simulations where the toxicity index was varied, in 80% of simulations where the study quality weights were varied, and in 20% of simulations where all input parameters were varied. The second highest hazard ranking order is permutation (c), Ag, TiO_2_, and ZnO. Significant sensitivity of the ranking order to changes in the LOE-specific toxicity index is highlighted by the relative uniformity of the ranking distributions across the permutations (a)–(e).

## 4. Discussion

This comparative study investigated the efficacy of quantitative WoE and Bayesian methodologies in predicting the hazard potential of metal and metal-oxide. The BN and WoE models used the same reference database to generate relative hazard rankings of TiO_2_, Ag, and ZnO. The results indicate that, while the relative hazard ranking remain consistent across both models (ZnO, Ag, TiO_2_; from highest hazard to lowest hazard), significant variability was observed when evaluated for stability and predictive accuracy. The ranking order from the WoE model was stable for 44% of 40,000 sampling scenarios with stressed input parameters. Cross-validation of the BN demonstrated 67% prediction accuracy overall, with significant variation amongst the NMs: TiO_2_ (54%), Ag (70%), ZnO (100%).

Both methodologies exhibit potential to support the comprehensive human health risk assessment for NMs. The methods allow for the incorporation of expert judgement to bridge the gap where experimental data is lacking, and to update hazard predictions as new information becomes available. While expert elicitation methods form the basis of each model’s construction, the incorporation of data to form a conclusion differs. The BN refines both its NM hazard probabilistic forecasts and causal interdependencies between variables (model parameters) through the application of machine learning techniques on the database. In contrast, the quantitative WoE model refines its “hazard score” every time a new study, or line of evidence, is evaluated according to the pre-determined criteria and metrics. Therein lies a significant advantage of the BN over the WoE model. For the BN, the model is created and adapts to the input data, whereas the scoring criteria of the WoE model remains constant.

The BN and WoE models both utilize physico-chemical, toxicological, and study type data to infer the hazard potential of TiO_2_, Ag, and ZnO. However, each experimental result contributes to the resulting hazard prediction equally within the BN framework. This is a limitation of the model as it neglects the relevance, quality, and reliability of the characterization experiments used within each study. Given that the toxicity of NMs is a complex function of several properties that are experimentally problematic to characterise, the inclusion of study quality criterion is important for a reliable hazard assessment tool. The quantitative WoE methodology controls the influence of each LOE on the final hazard score by weighting it according to study quality criteria.

A combination of both WoE and BN models would overcome the limitations described. Here, the WoE would evaluate the experimental evidence available according to a set of rules for accepting or rejecting evidence. This filtered evidence could then be used to train the BN, which, at this point, should be much more reliable.

## 5. Conclusions

Responsible innovation requires safety protocols to be integrated prior to the commercialization phase of any manufactured NM [38]. The proliferation of nano-enabled products has continued and this suggests the implicit acceptance on the part of employers of the potential hazard, exposure, and risk of nanomaterials [39]. The global market for manufactured nanomaterials was valued at $7.3 billion in 2016 and is projected to expand to $16.8 billion by 2022 [40]. This rapid advancement combined with the ambiguity of risk intelligence may result in many employers insufficiently reducing, controlling, or transferring the risk, and hence, neglecting to adequately protect their workers. Therefore, the establishment of appropriate human health risk assessment (RA) methodologies and tools are considered crucial to the sustainable development and application of NMs. Hazard identification, effects assessment, exposure assessment, and risk characterisation comprise the elements of a comprehensive RA framework for chemicals [5].

Quantitative models for manufactured NM hazard screening enable proactive risk minimization strategies in the design and development phase of NM production [14,41]. Researchers can ex-ante predict the impact of varying physico-chemical properties on the resulting hazard potential, thus promoting the safety-by-design principle of NM manufacturing. The BN model allows for this probabilistic forecasting as illustrated in Table 4 and Table 5.

In a human health context, hazard identification involves inferring substance-specific biological adverse effects from experimental (in vitro, in vivo data, in silico) observations [14,42]. Toxicological studies provide the relevant criteria for hazard determination. However, studies have revealed that size and physico-chemical properties of NMs induce unique or more aggressive biological activity at the nanoscale [43]. Physico-chemical characteristics of nanomaterials known to influence toxicity are surface area [44,45], surface coating [46], composition [45], purity, shape [47], primary particle size [48], aggregation [45], and crystal structure [49].

Dose-response assessment quantitatively determines the relationship between adverse effects (i.e., hazards) and a concentration of a substance in a controlled environment. Significant correlations between dose and biologically relevant endpoints or biomarkers are therefore utilized to determine no-observed-adverse-effect levels (NOAELs) and human health exposure thresholds, such as recommended exposure limits (RELs) or occupational exposure limits (OELs). Exposure scenario analysis subsequently forecasts the extent to which potentially vulnerable parties, such as factory workers, are exposed to material concentrations during the life cycle of a NM. By comparing predictions of scenario-based to threshold limits determined toxicologically, an explicit risk characterisation may be ascertained and used to inform strategic risk management decisions [41].

Control banding (CB) or risk matrices have been proposed as an appropriate framework to illustrate and measure the risk of NM to human health [50]. These tools determine the inherent risk posed by an NM through the product of exposure and hazard metrics. Despite numerous implementations of CB to assess the occupational risk NM [51,52], the preceding requirement of validated and transparent quantitative hazard and exposure ranking methods has not yet been conclusively fulfilled. Each methodology (e.g., hazard ranking, exposure prediction) must be scientifically evaluated in isolation due to the complexities posed by NM. The BN and WoE hazard screening methods implemented in this paper are fitting candidates for the hazard axis. However, a combination of WoE that would weight the quality of evidence data along with the BN may prove to be optimal hazard assessment framework.

## Figures and Tables

**Table 1:** Quantitative weight of evidence (WoE) results for nano-TiO_2_. Each line of evidence (LOE) represents experimental evidence from academic literature evaluated based on physico-chemical properties, toxicity, and study quality. The overall hazard of nano-TiO_2_ (V) is derived from sum of all LOE-specific hazard scores ($WI_{j}$).

ID (j)	Reference	LOE Index Values Based on Physico-Chemical Properties ($S_{j}^{p.chem}$)	LOE Index Values Based on Toxicity ($S_{j}^{tox}$)	Total LOE Index Values ($S_{j}$)	Study Quality Weight ($W_{j}$)	Normalised Study Quality Weight ($w_{j}$)	Weighted LOE Index Values ($WI_{j}$)
1	Baisch et al. [24]	41.67	87.50	73.75	0.61	0.04	3.24
2	Baisch et al. [24]	41.67	75.00	65.00	0.84	0.06	3.91
3	Baisch et al. [24]	50.00	75.00	67.50	0.84	0.06	4.06
4	Catalan et al. [25]	38.89	37.50	37.92	0.71	0.05	1.94
5	Catalan et al. [25]	38.89	62.50	55.42	0.79	0.06	3.13
6	Catalan et al. [25]	38.89	37.50	37.92	0.65	0.05	1.78
7	Chen et al. [26]	30.56	75.00	61.67	0.48	0.03	2.14
8	Duan et al. [27]	44.44	25.00	30.83	0.47	0.03	1.04
9	Duan et al. [27]	44.44	25.00	30.83	0.32	0.02	0.71
10	Farcal et al. [28]	61.11	25.00	35.83	0.77	0.06	1.99
11	Farcal et al. [28]	47.22	37.50	40.42	0.76	0.05	2.20
12	Fisichella et al. [29]	30.56	12.50	17.92	0.52	0.04	0.66
13	Fisichella et al. [29]	38.89	12.50	20.42	0.56	0.04	0.81
14	Gurr et al. [30]	33.33	62.50	53.75	0.50	0.04	1.94
15	Gurr et al. [30]	33.33	37.50	36.25	0.50	0.04	1.31
16	Hu et al. [31]	47.22	62.50	57.92	0.54	0.04	2.23
17	Leppanen et al. [32]	41.67	12.50	21.25	0.62	0.04	0.95
18	Lindberg et al. [33]	41.67	0.00	12.50	0.63	0.05	0.57
19	Lindberg et al. [33]	41.67	50.00	47.50	0.56	0.04	1.91
20	Shimizu et al. [34]	33.33	62.50	53.75	0.76	0.05	2.93
21	Tassinari et al. [35]	52.78	12.50	24.58	0.56	0.04	0.98
22	Wang et al. [36]	41.67	62.50	56.25	0.94	0.07	3.80
						Hazard Score (V)=44.24	

**Table 2:** Sample (15 cases) results of out-of-sample validation test for BN.

Case	Test Data											NM Hazard	
	Shape	Nanop-Article	Dissolution	Surface Area (m^2^/g)	Surface Charge (mV)	Surface Coatings	Surface Reactivity	Aggregation	Particle Size (nm)	Administration Route	Study Type	Actual	Predicted
1	Irregular	TiO_2_	0–25%	51–101.25	from −50 to −25	Silianes-aluminium	Low	High	10–50	-	In vitro	None	None
2	Amorph	TiO_2_	-	-	-	-	-	-	>100	Injection	In vivo	High	Medium
3	Sphere	TiO_2_	-	-	-	AHPP	-	Low	10–50	-	In vitro	None	None
4	Irregular	TiO_2_	-	15–51	-	-	-	High	>100	Oral	In vivo	None	Medium
5	Irregular	TiO_2_	-	51–101.25	from −50 to −25	Hydroxyl	-	Medium	50–100	Oral	In vivo	None	Low
6	Sphere	Ag	-	-	-	-	-	-	10–50	Inhalation	In vivo	High	High
7	Sphere	Ag	-	-	-	PVP	-	Low	50–100	Inhalation	In vivo	High	Medium
8	Sphere	Ag	-	-	-	-	-	-	10–50	Intravenous	In vivo	None	None
9	Sphere	Ag	-	-	-	Citrate	-	-	10–50	Oral	In vivo	Medium	Medium
10	Sphere	Ag	-	0–15	from −50 to −25	PVP	-	High	10–50	-	In vitro	None	Low
11	Sphere	Ag	0–25%	-	-	-	-	Low	10–50	Oral	In vivo	Medium	Medium
12	Sphere	Ag	-	-	0–25	-	-	Low	10–50	Oral	In vivo	High	Medium
13	Elongated	ZnO	-	0–15	0–25	None	-	Medium	>100	-	In vitro	High	High
14	Elongated	ZnO	0–25%	15–51	-	Triethoxycapryl silane	-	Medium	>100	-	In vitro	High	High
15	Irregular	ZnO	0–25%	-	-	-	Low	-	10–50	-	In vitro	High	High

**Table 3:** Sensitivity analysis of BN model. Entropy reduction indicates the degree to which NM hazard was sensitive to each input nodes of the model. Higher values signify higher sensitivity of the NM hazard mode to the input node.

Input Variable	Nanomaterial		
	TiO_2_	Ag	ZnO
Surface coatings	0.26	0.53	0.01
Surface area	0.22	0.26	0.02
Particle size	0.28	0.13	0.05
Surface charge	0.08	0.37	0
Aggregation	0.09	0.22	0.01
Shape	0.26	0	0
Surface reactivity	0	0	0.16
Dissolution	0	0	0
Administration route	0.19	0.64	0
Study type	0.34	0.07	0.02

**Table 4:** Effect of particle size on the normalised NM hazard potential for TiO_2_, Ag, and ZnO.

Particle Size	Nanomaterial Hazard Potential		
	TiO_2_	Ag	ZnO
from 0 to 10	100%	50%	86%
from 10 to 50	25%	58%	94%
from 50 to 100	42%	55%	100%
>100	73%	77%	89%
No Evidence	34%	61%	91%

**Table 5:** Effect of surface area on the normalised NM hazard potential for TiO_2_, Ag, and ZnO.

Surface Area	Nanomaterial Hazard Potential		
	TiO_2_	Ag	ZnO
from 0 to 15	56%	54%	94%
from 15 to 51	71%	58%	89%
from 51 to 101.25	28%	27%	88%
from 101.25 to 189	73%	4%	67%
from 189 to 2025	15%	92%	100%
No Evidence	34%	61%	91%

**Table 6:** Results of Monte Carlo sensitivity analysis for quantitative WoE methodology displaying the mean, standard deviation, and average absolute difference of the total weighted index value (*V*) from 10,000 simulations for each uncertainty scenario proposed; (i) variation of physico-chemical input parameters, (ii) variation of toxicity parameters, (iii) variation of study weight parameters, and (iv) variation of all (i)–(iii) parameters.

Nanomaterial	Parameter	Variation of Input Parameters			
		$S_{j}^{p.chem}$	$S_{j}^{tox}$	$W_{j}$	$S_{j}^{p.chem}$, $S_{j}^{tox}$, and $W_{j}$
TiO_2_ V=44.2	Mean (Standard Deviation)	46.6 (1.9)	47.6 (4.4)	42.7 (2.2)	49.9 (5.4)
	Average Absolute Deviation	2.6	4.5	2.2	6.5
Ag V=45.3	Mean (Standard Deviation)	47.7 (2.1)	48.4 (4.9)	45.4 (2.3)	50.0 (6.2)
	Average Absolute Deviation	3.3	4.7	1.9	6.3
ZnO V=52.3	Mean (Standard Deviation)	53.6 (4.4)	48.6 (10.3)	52.7 (0.7)	49.8 (12.5)
	Average Absolute Deviation	3.7	8.8	0.7	10.3

**Table 7:** Distribution of the hazard ranking order of nanoparticles resulting from Monte Carlo uncertainty analysis varying input parameters: (i) physico-chemical properties, (ii) toxicity potential, (iii) study weights, and (iv) all input parameters.

Alternative Orders	Rank from Lowest (1) to Highest (3) Hazard			Ranking % by Variations of Input Parameters				Total
	1	2	3	$S_{j}^{p.chem}$	$S_{j}^{tox}$	$W_{j}$	$S_{j}^{p.chem}$, $S_{j}^{tox}$, and $W_{j}$	
a	TiO_2_	Ag	ZnO	55%	22%	80%	20%	44%
b	TiO_2_	ZnO	Ag	6%	11%	0%	9%	7%
c	Ag	TiO_2_	ZnO	31%	20%	20%	21%	23%
d	Ag	ZnO	TiO_2_	2%	8%	0%	10%	5%
e	ZnO	TiO_2_	Ag	3%	21%	0%	21%	11%
f	ZnO	Ag	TiO_2_	2%	17%	0%	19%	10%

## Supplementary Materials

Supplementary materials can be found at http://www.mdpi.com/1422-0067/19/3/649/s1.

## Abbreviations

NM	nanomaterial
BN	Bayesian network
CPT	conditional probability table
CNS	central nervous system
OEL	occupational exposure limit
REL	recommended exposure limit
WoE	weight of evidence
MCDA	multi-criteria decision analysis
CB	control banding
WS	weighted sum
VOI	value of information
NM	nanomaterial

## Appendix C

**Table A1:** Quantitative WoE results for nano-Ag. Each LOE represents experimental evidence from academic literature evaluated based on physico-chemical properties, toxicity, and study quality. The overall hazard of nano-Ag (V) is derived from sum of all LOE-specific hazard scores ($WI_{j}$).

ID	Reference	LOE Index Values Based on Physico-Chemical Properties ($S_{j}^{p.chem}$)	LOE Index Values Based on Toxicity ($S_{j}^{tox}$)	Total LOE Index Values ($S_{j}$)	Study Quality Weight ($W_{j}$)	Normalised Study Quality Weight ($w_{j}^{\prime }$)	Weighted LOE Index Values ($WI_{j}$)
1	Braakhuis et al. [48]	47.22	62.50	57.92	0.84	0.07	4.25
2	Braakhuis et al. [48]	33.33	12.50	18.75	0.74	0.06	1.21
3	Braakhuis et al. [48]	47.22	12.50	22.92	0.60	0.05	1.21
4	Braakhuis et al. [53]	41.67	25.00	30.00	0.72	0.06	1.90
5	Braakhuis et al. [53]	36.11	62.50	54.58	0.71	0.06	3.41
6	Braakhuis et al. [53]	33.33	62.50	53.75	0.71	0.06	3.36
7	Braakhuis et al. [53]	33.33	12.50	18.75	0.66	0.01	0.20
8	Gaiser at al. [54]	47.22	75.00	66.67	0.79	0.07	4.60
9	Gaiser at al. [54]	47.22	75.00	66.67	0.63	0.05	3.65
10	Haberl et al. 2013 [55]	38.89	75.00	64.17	0.60	0.05	3.39
11	Lankveld et al. 2010 [56]	44.44	37.50	39.58	0.47	0.04	1.63
12	Lee et al. 2013 [57]	52.78	62.50	59.58	0.73	0.06	3.83
13	Loeschner et al. 2011 [58]	52.78	37.50	42.08	0.48	0.04	1.77
14	Nymark et al. 2013 [59]	58.33	50.00	52.50	0.58	0.05	2.67
15	Van der Zande et al. 2012 [60]	44.44	25.00	30.83	0.61	0.05	1.66
16	Van der Zande et al. 2012 [60]	61.11	25.00	35.83	0.61	0.05	1.93
17	Yun at al. 2015 [61]	44.44	62.50	57.08	0.92	0.08	4.59
						Hazard Score (V)=45.26	

## Appendix D

**Table A2:** Quantitative WoE results for nano-ZnO. Each LOE represents experimental evidence from academic literature evaluated based on physico-chemical properties, toxicity, and study quality. The overall hazard of nano-ZnO (V) is derived from sum of all LOE-specific hazard scores ($WI_{j}$).

ID	Reference	LOE Index Values Based on Physico-Chemical Properties ($S_{j}^{p.chem}$)	LOE Index Values Based on Toxicity ($S_{j}^{tox}$)	Total LOE Index Values ($S_{j}$)	Study Quality Weight ($W_{j}$)	Normalised Study Quality Weight ($w_{j}^{\prime }$)	Weighted LOE Index Values ($WI_{j}$)
1	Farcal et al. [28]	50.00	50.00	50.00	0.76	0.29	14.63
2	Farcal et al. [28]	52.78	50.00	50.83	0.76	0.29	14.87
3	Lu et al. [62]	38.89	62.50	55.42	0.59	0.23	12.68
4	Zhang et al. [63]	36.11	62.50	54.58	0.48	0.19	10.15
						Hazard Score (V)=52.34	
